# Fortbildung von Allgemein- und Viszeralchirurgen in der lebensrettenden Notfallchirurgie: Ergebnisse einer Umfrage unter Operationskursteilnehmern

**DOI:** 10.1007/s00104-020-01170-2

**Published:** 2020-04-20

**Authors:** C. Güsgen, F. Anger, T. Hauer, A. Willms, H. J. Buhr, C.-T. Germer, R. Schwab, J. F. Lock, Chirurgische Arbeitsgemeinschaft Militär- und Notfallchirurgie (CAMIN) der Deutschen Gesellschaft für Allgemein- und Viszeralchirurgie (DGAV)

**Affiliations:** 1 Klinik für Allgemein- Viszeral- und Thoraxchirurgie, Bundeswehrzentralkrankenhaus Koblenz, Koblenz, Deutschland; 2 Klinik und Poliklinik für Allgemein‑, Viszeral‑, Transplantations‑, Gefäß- und Kinderchirurgie, Universitätsklinikum Würzburg, Würzburg, Deutschland; 3 Klinik für Allgemein- und Viszeralchirurgie, Bundeswehrkrankenhaus Berlin, Berlin, Deutschland; 4 Deutsche Gesellschaft für Allgemein- und Viszeralchirurgie, Berlin, Deutschland

**Keywords:** Damage control surgery, Terroranschlagtrauma, Abdominaltrauma, Messerstichverletzung, Schussverletzung, Damage control surgery, Terror attack trauma, Abdominal trauma, Stab wounds, Shooting injuries

## Abstract

**Hintergrund:**

Die geringe Anzahl operativ zu versorgender Körperhöhlenverletzungen erfordert ein Umdenken in der chirurgischen Aus- und Weiterbildung. Ein entsprechendes Kursformat wird seit 2014 über die DGAV angeboten. Um Berechtigung, Bedarf, Nutzen und Erfolg eines solchen Kursformates zu erheben, erfolgte eine Evaluation durch die bisherigen Kursteilnehmer.

**Material und Methoden:**

Kursevaluation und zusätzliche Onlinebefragung der bisherigen Kursteilnehmer hinsichtlich Alter, Geschlecht, Ausbildungsstand, Fachrichtung, Versorgungsstufe des Krankenhauses, notfallchirurgischer Erfahrungen, der Häufigkeit chirurgischer Notfallversorgungen, Teilnahme an anderen Kursformaten, Erfahrungen nach der Kursteilnahme, Einschätzung der aktuellen Fort- und Weiterbildungssituation und Finanzierung solcher Kurse.

**Ergebnisse:**

Insgesamt 142 Kursteilnehmer evaluierten ihre Kursteilnahme, zusätzlich beantworteten 83 den Onlinefragebogen. Über 90 % berichteten von einem nachhaltigen positiven Einfluss des Kurses auf ihr notfallchirurgisches Handeln. Mehr als die Hälfte konnte von konkreten Notfallsituationen berichten, die sie aufgrund der Kursteilnahme besser bewältigen konnten. In der Notfallversorgung erfahrene Chirurgen bewerteten den eigenen Lernerfolg durch die Kursteilnahme signifikant häufiger positiv als ihre weniger erfahrenen Kollegen. Keinen Einfluss auf den Lernerfolg hatten eine Ober- oder Chefarztposition, die Versorgungsstufe des Krankenhauses, das Alter oder Geschlecht der Teilnehmer. Die Mehrheit der antwortenden Chirurgen befürwortet die Integration eines solchen Kursformates in die chirurgische Weiterbildung und fordert hierzu eine finanzielle Unterstützung.

**Schlussfolgerung:**

Kursformate, in denen notfallchirurgische Strategien und Fähigkeiten vermittelt werden, sind etabliert und werden sehr positiv evaluiert. Die Fort- und Weiterbildung in notfallchirurgischen Fähigkeiten und Kenntnissen liegt im gesellschaftlichen Interesse und zumindest anteilig auch in ihrer Verantwortung.

## Abstract (en)

**Background:**

Due to a decreasing number of emergency procedures for body cavity injuries, surgical training is inadequate and current educational concepts must be reconsidered. The German Society for General and Visceral Surgery has set up a surgical training course in 2014 to overcome this shortage. In order to assess the eligibility, needs, benefits and success of such a training format, participants were asked to evaluate the program.

**Material and methods:**

All participants evaluated the course during participation and were later asked to answer an online survey regarding their age, gender, level of surgical education, surgical discipline, level of care of the hospital, emergency surgical experience and frequency of performing emergency surgery, participation in other programs, experiences after participating in the course, rating of the current training curriculum and funding of such courses.

**Results:**

Out of 142 participants 83 replied to the online survey. Over 90% reported a lasting positive influence of the course on emergency surgical skills. More than half of the responders remembered a clinical situation which they successfully managed due to the skills they gained during the course. Surgeons experienced in emergency treatment felt significantly more benefit than less experienced colleagues. A consultancy position, the level of care of the hospital, age and sex of the participants did not influence the overall benefits reported. The majority of responding surgeons were in favor of including such a training course in surgical education and stipulated public financial support.

**Conclusion:**

Course formats that mediate emergency surgery strategies and skills are established and well accepted. Training of surgeons in life-saving emergency surgery is in the public interest and is also partly the responsibility of society.

## Hintergrund

Die Schlüsselmanöver der lebensrettenden Notfallchirurgie sind primär die schnellst mögliche Blutstillung und sekundär die Kontrolle von Kontaminationen. Das letale Potenzial ist bei Blutungen in die Körperhöhlen, Thorax und Abdomen sowie an den Übergängen des Körperstamms zu den Extremitäten am höchsten [5]. Während sich äußerlich sichtbare Blutungen der Extremitäten durch die Anlage eines Tourniquets (temporär) stoppen lassen, ist die Versorgung von Blutungen in den Körperhöhlen oder an den stammnahen Übergängen zu den Extremitäten nur unter Klinikbedingungen zu erreichen. Grundvoraussetzungen zur erfolgreichen innerklinischen Versorgung einer Höhlenverletzung ist ein chirurgisches Team, das über die erforderliche Kompetenz der notfallchirurgischen thorakoabdominellen Versorgungstechniken verfügt. Die geringe Anzahl operativ zu versorgender Körperhöhlenverletzungen erfordert ein Umdenken in der chirurgischen Aus- und Weiterbildung.

### Aktuelle Weiterbildungssituation in Deutschland

Ein Problem liegt in der 2005 geänderten und 2006 umgesetzten Weiterbildungsordnung (WBO) für den Erwerb der verschiedenen chirurgischen Facharztdisziplinen [13, 14]. Durch den Wegfall der zuvor obligaten Weiterbildung zum Allgemeinchirurgen wurde, nach 2 Jahren Basischirurgie, eine deutlich frühere Spezialisierung ermöglicht. In der Folge konzentrierten sich die Vorgaben notfallchirurgisch zu erlangender operativer Fähigkeiten im Bereich der Körperhöhlen auf die Allgemein- und Viszeralchirurgie.

Da vor allem die Therapiekonzepte des stumpfen Abdominaltraumas einem Wandel unterliegen, wird die Entscheidungsfindung zwischen konservativen, interventionellen und operativen Therapiemöglichkeiten nach den Empfehlungen der S3-Leitlinie Polytrauma zunehmend komplexer [6]. Sie stellt damit höhere Ansprüche an die Erfahrung und Expertise des therapieentscheidenden Chirurgen. Nach Daten des Traumaregisters der Deutschen Gesellschaft für Unfallchirurgie (TraumaRegister DGU®) erleiden ca. 15–20 % der polytraumatisierten Patienten ein Abdominaltrauma [4]. Im Fall stumpfer Verletzungsmechanismen wurde die Indikation zur konservativen oder interventionellen Versorgung deutlich ausgeweitet, was die Zahl der abdominellen Notfalloperationen weiter reduziert hat.

Penetrierende abdominelle Verletzungen sind zwar nahezu immer operativ zu versorgen, stellen aber mit ca. 5 % aller polytraumatisierten Patienten eine relativ kleine Gruppe [4]. Leidvolle Erfahrungen bez. terroristischer Anschläge der letzten Jahre in Europa und auch in Deutschland haben einen Wandel im Bewusstsein hervorgebracht und den Bedarf einer qualifizierten chirurgischen Terrorvorbereitung gezeigt [9, 19]. Dabei rückt speziell die chirurgische Expertise der penetrierenden Körperhöhlenverletzung in den Fokus [10].

### Grundlage des DGAV-Operations-Workshops „Viszeralchirurgischer Notfall und thorakoabdominelles Trauma“

Die Frage, inwiefern notfallchirurgische Fähigkeiten unter den aktuellen Alltagsbedingungen mit einer derart geringen Anzahl an entsprechenden Patienten erworben und aufrecht gehalten werden können, lässt sich nur durch Simulation im Rahmen notfallchirurgischer Kursformate beantworten. Ein Kursformat wird seit 2014 über die DGAV, speziell über ihre Arbeitsgemeinschaft CAMIN (Chirurgische Arbeitsgemeinschaft für Militär- und Notfallchirurgie) angeboten. Um Berechtigung, Bedarf und Nutzen eines solchen Kursformates zu erheben, erfolgte eine Evaluation durch die bisherigen Kursteilnehmer.

## Methodik

### DGAV/CAMIN-Operations-Workshop

Das Konzept des Kurses wurde im Zeitraum 2013 bis 2014 von den Autoren erarbeitet. Es wurde kurrikular für Viszeralchirurgen entwickelt. Die Zielvorgaben bestanden in der Vermittlung theoretischer Grundlagen mit Sensibilisierung eines trauma- bzw. verletzungsphysiologischen „mindset“ sowie praktischer und realitätsnaher Übung und Umsetzung effektiver notfallchirurgischer Techniken (Skills) im Thorax und Abdomen. Auf der Grundlage der täglich erlebten elektiven und hochspezialisierten Viszeralchirurgie wurde auch der viszeralchirurgische Notfall im Kurs thematisiert. Insgesamt wurde ein dreiteiliges Konzept aus Vorträgen, anatomischen Präparationen an Körperspendern und Operationen im narkotisierten Großtiermodell (Live-tissue-Modell) mit jeweils gleichen Zeitanteilen erarbeitet.

In den Vorträgen wurden die Themen thorakoabdominelles Trauma, Terroranschlagtrauma, Grundlagen der „damage control surgery“ und Traumaphysiologie, temporäre und definitive Blutungskontrolle, temporäre Laparostomaanlage, Anastomoseninsuffizienz und Peritonitistherapie, Stomaanlage sowie gastrointestinale Blutungen erörtert.

Die anatomischen Präparationen fokussierten zunächst auf die unterschiedlichen Zugangswege zur raschen Blutungskontrolle bei Thorax‑, Abdomen- und Verletzungen der Körperübergänge sowie Extremitäten mit strukturierter Anleitung für die Exposition und Kontrolle kritischer Gefäße wie Aorta abdominalis mit sämtlichen viszeralen Ästen, die Aorta thoracalis, die Vena cava inferior, Pfortader, A./V. subclavia, A./V. femoralis und V. saphena magna.

Im Großtiermodell wurden realitätsnah die Traumalaparotomie, abdominales Tamponieren und systematische Exploration, Kocher‑, Catell-Braasch‑, Mattox-, und Pringle-Manöver sowie Damage-control-surgery-Prozeduren wie Splenektomie, Darmnaht und -resektion, Leberpacking, Blutstillungstechnik, Pankreasdrainage, Nephrektomie, Zwerchfellnaht, Thorakotomie, Lungen- und Herznaht sowie einfache Gefäßrekonstruktionen inkl. temporäres Shunting praktisch geübt.

Sämtliche Tieroperationen wurden durch die verantwortlichen Behörden genehmigt und im Einklang mit dem deutschen Tierschutzgesetz sowie dem europäischen Tierschutzrecht durchgeführt. Alle Kursinhalte wurden in einen 2‑tägigen Intensivkurs mit einer maximalen Teilnehmerzahl von 16 Chirurgen integriert. Insgesamt wurden im Zeitraum 11/2014 bis 05/2019 10 Kurse am Universitätsklinikum Würzburg und ein Kurs am Universitätsklinikum Düsseldorf durchgeführt. Nach 7 erfolgreichen Kursen unter dem Titel „Viszeralchirurgischer Notfall und thorakoabdominelles Trauma“ erfolgte eine Überarbeitung des Kurrikulums. Durch Integration des ASSET™(Advanced Surgical Skills for Exposure in Trauma)-Moduls erhielt der Kurs eine internationale Zertifizierung durch das American College of Surgeons gemäß den ASSET™-Regularien. Das ASSET™-Kursmodul vermittelt algorithmisch die systematische, chirurgische Gefäßexposition an Hals, Thorax, Abdomen und den Extremitäten zur Blutstillung nach Verletzung. Zwischen 2017 bis 2019 wurden 4 Kurse unter dem Titel „Zugänge und Techniken des thorakoabdominellen Traumas – viszeralchirurgische Notfälle inkl. ASSET™-Kurs“ durchgeführt.

### Umfrage

Im Zeitraum zwischen 2014 und 2019 nahmen insgesamt 142 Teilnehmer an dem DGAV-Operations-Workshop teil. Dabei erfolgte die Standardkursevaluation der DGAV primär unmittelbar am Ende des Kurses durch jeden Teilnehmer. Es wurden nun alle bisherigen Kursteilnehmer um Beantwortung einer Onlineumfrage gebeten. Die Teilnahme an der Umfrage erfolgte freiwillig, unentgeltlich und anonym. Primäres Ziel der Umfrage war es, positive oder negative Effekte der Kursteilnahme im klinischen Arbeitsalltag der Teilnehmer zu erfragen, Änderungswünsche zu identifizieren und die Sinnhaftigkeit eines solchen Kursformates im zeitlichen Intervall zu überprüfen.

Zur Messung subjektiver Einschätzungen wurden Aussagen formuliert und mit einer 5‑stufigen Likert-Skala abgefragt. Insgesamt wurden 24 Fragen formuliert:

- Angaben zu Alter, Weiterbildungsstand, Tätigkeit, Erfahrung (9 Fragen),
- persönliche Erfahrungen nach der Kursteilnahme (7 Fragen),
- Einschätzungen zur chirurgischen Fortbildung in der Notfall‑, Trauma- und Katastrophenchirurgie (8 Fragen).

Die Erstellung des Fragebogens und die Onlineumfrage wurden mit EvaSys Survey Automation Suite erstellt und ausgewertet (Electric Paper Evaluationssysteme GmbH, Lüneburg, Deutschland).

### Statistische Auswertung

Deskriptive Daten werden als Median und Intervall, absolute Zahlen als Gesamtzahl mit Prozent (soweit nicht anders dargestellt) angegeben. Die statistische Analyse erfolgte mittels χ^2^- oder Fishers Exakt-Test in Abhängigkeit der Datenskalierung und -verteilung. Das Signifikanzniveau betrug *p* < 0,05 (zweiseitig). Die Analyse wurde unter der Verwendung von IBM SPSS Statistics Version 25 durchgeführt.

## Ergebnisse

### Kursteilnehmer und Umfrageteilnehmer

Informationen zu Alter, Geschlecht, Ausbildungsstand, Fachrichtung und der Tätigkeit nach Versorgungsstufe des Krankenhauses aller Kursteilnehmer sowie der Teilnehmer an der Umfrage sind in Tab. 1 angegeben. Die Mehrheit der Teilnehmer waren Fach- und Oberärzte für Allgemein- und Viszeralchirurgie aus Krankenhäusern der Grund- bis Schwerpunktversorgung.

**Table 1:** 

	Kursteilnehmer	Umfrageteilnehmer
*n (%)*	142 (100 %)	83 (58 %)
*Kursformat*		
Viszeralchirurgischer Notfall und thorakoabdominelles Trauma	91 (64 %)	49 (59 %)
Zugänge und Techniken des thorakoabdominellen Traumas – viszeralchirurgische Notfälle inkl. ASSET™	51 (36 %)	34 (41 %)
*Alter [Jahre]*	40 [28–63]	42 [33–65]
*Geschlecht*		
Männlich	94 (66 %)	53 (64 %)
Weiblich	48 (34 %)	30 (36 %)
*Berufliche Position*		
Assistenzarzt	21 (15 %)	6 (7 %)
Facharzt	50 (35 %)	24 (29 %)
Oberarzt	68 (48 %)	49 (59 %)
Chefarzt	3 (2 %)	4 (5 %)
*Versorgungsstufe*		
Grund und Regelversorgung	66 (46 %)	39 (47 %)
Schwerpunktversorgung	44 (31 %)	26 (31 %)
Maximalversorgung	26 (18 %)	12 (14 %)
Universitätsklinikum	6 (5 %)	6 (7 %)
*Fachrichtung*^a^		
Allgemeinchirurgie	75 (53 %)	19 (23 %)
Viszeralchirurgie	54 (38 %)	55 (66 %)
Andere Fachrichtung	13 (9 %)	9 (11 %)

^a^In der Kursevaluation wurden die Teilnehmer nach ihrer Facharztqualifikation, in der Onlineumfrage nach ihrem überwiegenden Tätigkeitsbereich befragt. Daher ergeben sich hier unterschiedlichen Angaben

**Figure 1:**
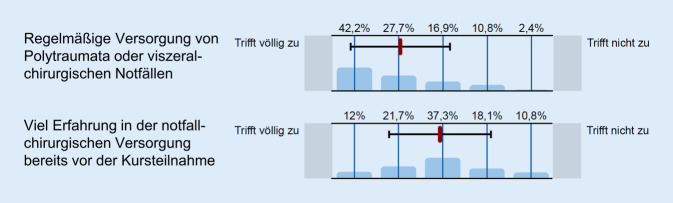

In der Onlineumfrage wurden die Kursteilnehmer gebeten, zusätzliche Angaben zu ihrer vorbestehenden Erfahrung und der Häufigkeit in der chirurgischen Notfallversorgung anzugeben (Abb. 1). Siebzig Prozent der Umfrageteilnehmer gaben an, regelmäßig polytraumatisierte Patienten und viszeralchirurgische Notfälle zu versorgen. Die Häufigkeit dieser Angabe war nicht von der Versorgungsstufe des Krankenhauses abhängig, in dem die Umfrageteilnehmer arbeiten (62 % in Grund- und Regelversorgung, 81 % in Schwerpunktversorgung, 75 % in Maximalversorgung, 67 % in Uniklinika, *p* = 0,4). Die Mehrheit der Umfrageteilnehmer (54 %) hatte bereits vor der Kursteilnahme viel Erfahrung in der selbständigen Notfallchirurgie und/oder bereits an Kursen im Bereich Notfall‑, Trauma- und Katastrophenchirurgie teilgenommen (41 % insgesamt, davon 35 % ATLS [Advanced Trauma Life Support], 11 % PHTLS [Prehospital Trauma Life Support], 7 % DSTC [Definitive Surgical Trauma Care], 7 % TDSC [Terror and Disaster Surgical Care], 5 % ETC [European Trauma Course]). Die Anzahl erfahrener Chirurgen aus den Kliniken der Maximalversorgung/Unikliniken war mit 83 % vs. 46 % Chirurgen aus Kliniken der Regel- bis Schwerpunktversorgung signifikant höher (*p* = 0,005). Drei Viertel der Teilnehmer waren Mitglieder der DGAV.

### Kursevaluation

**Figure 2:**
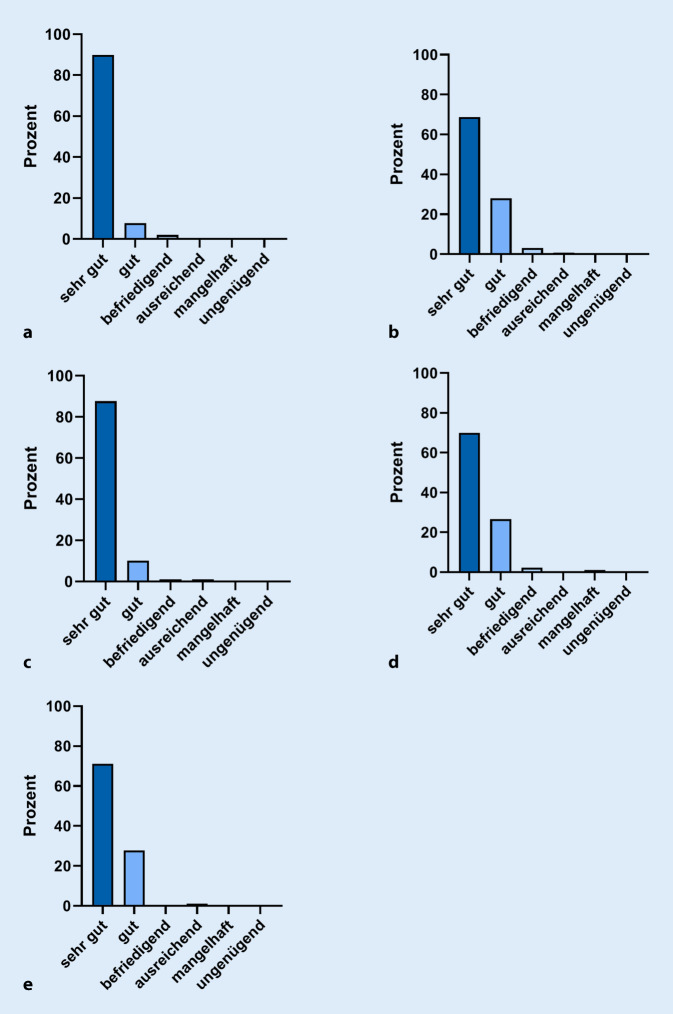

Die unmittelbar zum Ende des Kurses vollzählig abgegebenen Evaluationsbögen zeigten insgesamt eine sehr gute Bewertung des Kursformates (Abb. 2). Fast alle (95 %) Kursteilnehmer gaben an, den Kurs „uneingeschränkt“ und 5 % „eher“ weiterzuempfehlen. Die Kursgebühren wurden bei 52 % der Teilnehmer vollständig vom Arbeitgeber übernommen, 14 % erhielten eine Unterstützung, aber 35 % finanzierten die Kursteilnahme selbst. Die Kursgebühren von 1650 € wurden von 77 % der Teilnehmer als angemessen beurteilt, 17 % beurteilten den Kurs als zu teuer. Immerhin 84 % der Teilnehmer erhielten eine Freistellung vom Arbeitgeber, 12 % glichen Überstunden aus und 4 % nahmen Urlaubstage in Anspruch (Daten nicht gezeigt).

### Erfahrungen nach der Kursteilnahme

**Figure 3:**
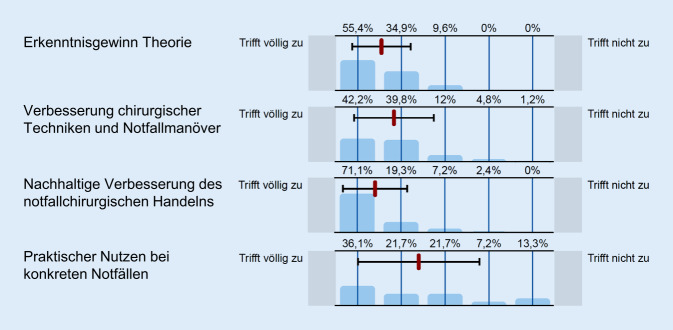

In der Onlineumfrage wurden die Chirurgen zu ihren klinischen Erfahrungen in der lebensrettenden Notfallchirurgie nach der Kursteilnahme befragt (Abb. 3). Nahezu alle Umfrageteilnehmer gaben einen Lernzuwachs im Bereich der theoretischen Inhalte (90 %) und praktischen Fertigkeiten (82 %) an. Über 90 % berichteten von einem nachhaltigen positiven Einfluss auf ihr notfallchirurgisches Handeln. Über die Hälfte der Teilnehmer (58 %) konnte von konkreten Notfallsituationen berichten, die sie aufgrund der Kursteilnahme erfolgreich bewältigen konnten.

Die subjektive Beurteilung des eigenen Lernerfolgs durch die Kursteilnahme unterschied sich statistisch signifikant zwischen notfallchirurgisch erfahrenen Kollegen und weniger erfahrenen Kollegen („sicherer in Theorie und Prinzipien“ 100 % vs. 79 %, *p* = 0,001; „sicherer in chirurgischen Manövern“ 91 % vs. 71 %, *p* = 0,018; „nachhaltig positiver Einfluss“ 98 % vs. 82 %, *p* = 0,013). Keinen statistisch signifikanten Einfluss auf den Lernerfolg hatten die berufliche Position (Fach- oder Oberarzt), die Versorgungsstufe des Krankenhauses, das Alter oder Geschlecht der Teilnehmer. Von konkreten Notfallsituationen konnten häufiger Oberärzte als Fach- oder Assistenzärzte berichten (66 % vs. 43 %, *p* = 0,04).

Wir baten die Teilnehmer um Nennung konkreter Situationen, bei denen sie von der Kursteilnahme besonders profitiert hatten. Hierzu gab es u. a. folgende Angaben:

- thorakale Messerstichverletzungen mit Verletzung der Vena cava,
- multiple Messerstichverletzungen thorakoabdominell,
- abdominelle Schussverletzungen mit hämorrhagischem Schock,
- Notfallnephrektomie bei traumatischer Nierenzerreißung,
- Notfallsplenektomie im hämorrhagischen Schock,
- temporäre abdominelle Blutungskontrolle mittels intraaortalem Ballonkatheter im Schockraum,
- Notfalllaparotomie bei abdomineller Arterienruptur im Angiographielabor,
- Femoralarterienblutung nach Koronarangiographie.

Außerdem baten wir darum, anzugeben, von welchen Kursanteilen die Teilnehmer am meisten profitiert hatten. Die Mehrheit (55 %) gab an, dass insbesondere die Kombination aus Theorie, Anatomie und Live-tissue-Modell besonders hervorzuheben sei. Neunundzwanzig Prozent profitierten am meisten vom Live-tissue-Modell, 12 % von der anatomischen Präparation, 4 % von den theoretischen Vorträgen.

### Einschätzungen der aktuellen Fortbildungssituation

**Figure 4:**
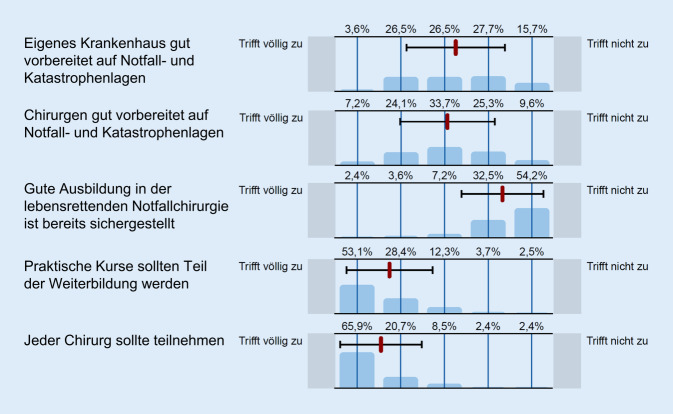

Die Vorbereitung auf besondere Notfall- oder Katastrophenlagen sowie lebensrettende Notfallchirurgie in den eigenen Krankenhäusern wurde von den Umfrageteilnehmern kritisch beurteilt (Abb. 4). Nur 30 % der Umfrageteilnehmer gaben an, ihr Krankenhaus sei gut vorbereitet. Es zeigte sich hier ein großer Unterschied entsprechend der Versorgungsstufe des Krankenhauses. Antwortende aus Krankenhäusern der Maximalversorgung oder Unikliniken sahen in 61 % eine gute Vorbereitung, wohingegen diese Einschätzung nur von 22 % der Umfrageteilnehmer aus Krankenhäusern der Grund- bis Schwerpunktversorgung geteilt wurde (*p* = 0,001).

Auch die Qualifikation der Chirurgen wurde zwischen den Versorgungsstufen unterschiedlich beurteilt. Hier beurteilten 56 % der Umfrageteilnehmer aus Krankenhäusern der Maximalversorgung oder Unikliniken die Qualifikation ihrer Kollegen positiv, während dies nur 25 % der Umfrageteilnehmer aus Krankenhäusern der Grund- bis Schwerpunktversorgung taten (*p* = 0,012).

Die bestehende Weiterbildung zum Allgemein- oder Viszeralchirurgen wurde von fast allen Antwortenden kritisch bez. des Erwerbs notfallchirurgischer Kompetenzen bewertet. Fast 90 % (87 %) gaben an, dass durch die aktuelle Weiterbildungsordnung keine adäquate Weiterbildung in diesem Bereich sichergestellt wäre. Diese Einschätzung zeigte sich durchgehend unabhängig von Erfahrung und Versorgungsstufe, wurde jedoch von den Chefärzten etwas positiver beurteilt.

Dementsprechend befürworteten 80 % der Umfrageteilnehmer, einen praktischen Kurs für Notfallchirurgie in die Weiterbildung zum Viszeralchirurgen zu integrieren. Auch diese Einschätzung zeigte sich unabhängig von notfallchirurgischer Erfahrung, Versorgungsstufe oder beruflicher Position. Die Frage wurde von allen Assistenzärzten bejaht. Außerdem gaben 86 % der Umfrageteilnehmer an, dass generell jeder Chirurg einen speziellen praktischen Kurs für lebensrettende Notfallchirurgie absolvieren sollte. Auch die Frage bezüglich der Sinnhaftigkeit eines Wiederholungs- oder Auffrischungskurses wurde von den Umfrageteilnehmern positiv beurteilt. Während nur 4 % eine wiederholte Kursteilnahme für nicht sinnvoll erachteten, wurde eine erneute Kursteilnahme nach 2 bis 3 Jahren (60 % der Befragten) bzw. nach 5 bis 10 Jahren (36 % der Befragten) als sinnvoll eingeschätzt.

### Finanzierung des Kurses

In der abschließenden Frage bez. der Finanzierung solcher Kursformate wünschten sich 59 % der Umfrageteilnehmer eine gemeinsame Finanzierung durch Bundesländer und Krankenhäuser, 19 % alleinig durch die Bundesländer und 19 % alleinig durch die Krankenhäuser.

## Diskussion

In der vorliegenden Arbeit konnten wir durch eine Onlinebefragung der bisherigen Kursteilnehmer eine sehr positive Langzeitbewertung des DGAV-Operations-Workshops „Viszeralchirurgischer Notfall und thorakoabdominelles Trauma“ nachweisen. Bei nahezu allen Antworten wurde ein Zugewinn an Sicherheit bei der strategischen und praktischen Umsetzung im Rahmen notfallchirurgischer Situationen und Eingriffe angegeben. Die innerklinischen Strukturen sowie die Qualifikation der Chirurgen zur Bewältigung notfallchirurgischer Szenarien im eigenen Krankenhaus wurden dagegen schlecht bewertet. Die Teilnehmer der Umfrage empfehlen daher, das beurteilte Kursformat in die Fortbildung aller Chirurgen, die in die Akutversorgung vital bedrohter Patienten involviert sind, zu integrieren. Dementsprechend wurde eine finanzielle Förderung solcher Kursformate aus öffentlichen Kassen stark befürwortet. Doch wie kommt es, dass die derzeitige Weiterbildungssituation derart kritisch beurteilt wurde?

### Aktuelle Vorgaben durch die jeweilige Weiterbildungsordnung (Tab. 2)

**Table 2:** 

Facharzt für	Erwerb von Kenntnissen, Erfahrungen und Fertigkeiten	Geforderte Anzahl von Notfalloperationen
*Allgemeinchirurgie*	… in der operativen Notfallversorgung bei gefäß-, thorax-, unfall- und viszeralchirurgischen einschließlich koloproktologischer Verletzungen	**–**
*Unfallchirurgie *[13]	… den zur Versorgung im Notfall erforderlichen Maßnahmen in interdisziplinärer Zusammenarbeit	*10* Notfalleingriffe, z. B. in Körperhöhlen
*Spez. Unfallchirurgie *[15]	… den zur Behandlung von Schwer- und Mehrfachverletzten erforderlichen Maßnahmen einschließlich mikrochirurgischer Techniken und des Traumamanagements in interdisziplinärer Zusammenarbeit	*25* Notfalleingriffe in Körperhöhlen
*Viszeralchirurgie *[14]	… der operativen und nichtoperativen Notfallversorgung bei viszeralchirurgischen einschließlich der koloproktologischen Verletzungen	*10* Notversorgung von Leber- und Milzverletzungen *30* Notfalleingriffe des Bauchraums
*Spez. Viszeralchirurgie* [16]	… der Erkennung und nichtoperativen sowie operativen Behandlung einschließlich der postoperativen Überwachung komplexerer Verletzungen innerer Organe	*15* Notfalleingriffe des Bauchraums *5* Eingriffe bei Abdominaltrauma

Die Weiterbildungsordnung (WBO) des Facharztes für Unfallchirurgie sieht für die Erlangung einer notfallchirurgischen Kompetenz auf dem Gebiet der Körperhöhlenverletzung z. B. 5 Tracheotomien und 5 Thoraxdrainagen vor. Die Milzverletzung des hämodynamisch instabilen, polytraumatisierten Patienten wird damit für beide Betroffene, den Operateur und den Patienten, zu einer nicht angebrachten Herausforderung. Darf also vom Facharzt für Unfallchirurgie, erworben nach aktueller WBO, eine Versorgungskompetenz hinsichtlich des Höhlentraumas erwartet werden?

In Kliniken der Maximalversorgung oder regionalen Versorgungsstufen wird diesem Umstand dadurch Rechnung getragen, dass in aller Regel sowohl Unfall- als auch Viszeralchirurgen im Rahmen der Polytraumaversorgung unmittelbar zur Verfügung stehen. Dies ist in Krankenhäusern niedrigerer Versorgungstufen außerhalb der regulären Arbeitszeiten häufig nicht möglich. So stuften die Umfrageteilnehmer aus Krankenhäusern der Grund- und Regelversorgung die notfallchirurgische Qualifikation lediglich in einem Viertel der Fälle als ausreichend ein. Aber auch an den Kliniken der Maximalversorgung wurde eine adäquate Qualifikation der Kollegen für die lebensrettende Notfallchirurgie nur in 56 % der Fälle geäußert.

Es verwundert deshalb nicht, dass die überwiegende Mehrheit der Befragten einen praktischen Kurs für Notfallchirurgie als festen Bestandteil der chirurgischen Weiterbildung befürworten. Das Kursformat ist in der Lage, aufgrund klar definierter Handlungsalgorithmen und strukturierter notfallchirurgischer Fertigkeiten, den Ansprüchen der Teilnehmer auch in der Kürze der Zeit gerecht zu werden. Dementsprechend gaben fast alle Befragten an, dass jeder Chirurg an einem praktischen Kurs für lebensrettende Notfallchirurgie teilnehmen sollte. Auch ein Wiederholungs- oder Auffrischungskurs wurde von den Befragten als sinnhaft erachtet.

Eine Limitation der Studie besteht in einem möglichen Selektionsbias, da Teilnehmer eines derartigen Kursformates bereits einen Bedarf in ihrer notfallchirurgischen Fortbildung perzipiert haben und eine Gruppe darstellen, die möglicherweise per se kritisch hinsichtlich ihrer persönlichen Qualifikation auf dem Gebiet der Notfallchirurgie ist. Dem entgegen steht aber, dass die Mehrheit der Teilnehmer bereits vor der Kursteilnahme viel Erfahrung in der selbständigen Notfallchirurgie hatte und/oder bereits an Kursen im Bereich Notfall‑, Trauma- und Katastrophenchirurgie teilgenommen hat. Die Notwendigkeit derartiger Kursformate zum Kompetenzaufbau notfallchirurgischer Techniken wird durch vielfache und weltweit angebotene Kursformate der chirurgischen Vereinigungen besonders hervorgehoben. Neben der regulären chirurgischen Weiterbildung wird der Kompetenzerwerb, teils modulähnlich, durch verschiedene Kursformate ermöglicht (Tab. 3).

**Table 3:** 

Kursname	Organisation	Kursorte
*Emergency surgery course (ESC®)*	ESTES (European Society for Trauma and Emergency Surgery) in Kooperation mit der American Association for the Surgery of Trauma	Europa
*Clinical Skills in Emergency Surgery*	Royal College of Surgeons (UK)	England
*Specialty Skills in Emergency Surgery and Trauma (SSET)* *Definitive Surgical Trauma Skills (DSTS)*	Royal College of Surgeons (UK)	England
*EASC – Emergency Abdominal Surgery Course*	WSES (World Society of Emergency Surgery)	Europa
*Definitive Surgical Trauma Care (DSTC®)*	IATSIC (International Association for Trauma Surgery and Intensive Care)	Amerika, Asien, Europa, Afrika
*Advanced Trauma Operative Management (ATOM™)*	Committee on Trauma des American College of Surgeons (ACS)	USA
*Thorakoabdominelles Trauma und viszeralchirurgischer Notfall*	DGAV (Deutsche Gesellschaft für Allgemein- und Viszeralchirurgie)/CAMIN	Deutschland
*Advanced Surgical Skills for Exposure in Trauma*	Committee on Trauma des American College of Surgeons (ACS)	USA, Europa

Eine weitere Limitation stellt die fehlende Validierung des geäußerten Zugewinns an Kompetenz und Sicherheit in der späteren chirurgischen Notfallversorgung durch die Kursteilnahme dar. Wenngleich 90 % der Kursteilnehmer einen nachhaltig positiven Einfluss auf ihr notfallchirurgisches Handeln angeben, konnten nur 58 % von konkreten Notfallsituationen berichten. In der aktuellen Literatur kann der Effekt dieser Kursformate aber eindrücklich reproduziert und damit bestätigt werden [1, 21].

### Lebensrettende Notfallchirurgie setzt Erfahrung voraus

Die Tatsache, dass drei Viertel der Kursteilnehmer zumindest Fachärzte, größtenteils sogar Oberärzte waren, zeigt den Fortbildungsbedarf auch und besonders bei erfahrenen Kollegen. Zwar wird im Anforderungsprofil des Kurses auf einen Facharzt- bzw. Oberarztstatus hingewiesen, dennoch wird hier deutlich, dass offenbar viele Chirurgen keine ausreichende notfallchirurgische Expertise während der Weiterbildung erworben haben und damit über keine ausreichende Handlungssicherheit im Notfall verfügen. Wenn 70 % der Kursteilnehmer angeben, regelmäßig entweder polytraumatisierte Patienten oder viszeralchirurgische Notfälle zu versorgen, unterstreicht dies die Relevanz der Thematik. Insbesondere, da im Fall eines Massenanfalls von Verletzten oder gar Terroranschlags, in der Regel die nächstgelegene Klinik aufgesucht wird [8, 12, 20]. Der Chirurg in ländlichen Regionen kann also durchaus mit der Situation konfrontiert werden, thorakoabdominelle Verletzungen erstversorgen zu müssen. Die Autoren weisen darauf hin, dass die Versorgung von Patienten mit thorakoabdominellem Trauma grundsätzlich in einem Krankenhaus der entsprechenden Versorgungsstufe durch erfahrene und spezialisierte Kollegen anzustreben ist. Die Versorgungssituation in Deutschland, die geringe Inzidenz des penetrierenden Traumas sowie die persistierende Bedrohung durch Attentate bzw. Anschläge verlangt nach entsprechender Ausbildung und Vorbereitung.

Algorithmusbasierte Kursformate der Schockraumversorgung bilden dabei den Grundstein für eine lebensrettende Therapie. Der Erfolg dieser Formate ist mittlerweile bewiesen [2, 17]. Auch die lebensrettenden, operativen Sofortmaßnahmen „beyond ATLS®“ lassen sich algorithmisch trainieren. Das wurde im evaluierten Kursformat konsequent umgesetzt.

Wichtige Voraussetzung hierzu ist das Nachvollziehen der theoretischen Herangehensweise mit klar definierten Prinzipien. So verfügt der erfahrene viszeralchirurgische Oberarzt über die entsprechenden operativen Fähigkeiten der Blutstillung in den Körperhöhlen, wird aber im elektivchirurgischen Alltag nur sehr selten mit der Indikationsstellung zu notfallchirurgischen Strategien wie den „Damage-control-surgery“-Verfahren konfrontiert. Die richtige Entscheidungsfindung kann beim einzelnen Patienten lebensrettend sein oder zusätzliche Morbidität verhindern. Neun von 10 Kursteilnehmern bewerten den Aufbau des 2‑tägigen Kurses mit seinen Inhalten als optimal. Die Kombination aus kurzen, auf das Wesentliche konzentrierten und kondensierten theoretischen Algorithmen, die Systematik der möglichen Gefäßzugänge im Rahmen des ASSET™-Kursteils sowie das Live-tissue-Training wird als sehr effektiv eingeschätzt [3, 7]. Im aktuellen Kursformat werden die verschiedenen Simulationsanteile als optimal aufeinander abgestimmt bewertet. In der Abfrage spezieller Techniken standen notfallchirurgische Versorgungen insbesondere penetrierender Verletzungen im Fokus. Hier konnte der Kurs für nahezu alle Kursteilnehmer relevante Sicherheit in der Versorgungskompetenz derartiger Verletzungen bringen. Einen langfristig positiven Effekt entsprechender Kursformate zeigten sich ebenfalls in den Evaluierungen eines norwegischen Operationskurses für „damage control surgery“ [11] sowie auch eines niederländischen Traumakurses [21].

Interessanterweise gaben die erfahrenen Chirurgen einen noch höheren Lernerfolg als die weniger erfahrenen Kollegen an. Dies ist aus unserer Sicht auf das anspruchsvolle Kursformat zurückzuführen. Es wurde primär für Chirurgen in Oberarztfunktion konzipiert, die die notwendigen chirurgischen Techniken im elektiven Bereich sicher beherrschen. Somit war es den erfahrenen Kollegen besser möglich, die Vielzahl der geübten „Damage-control-surgery“-Manöver unter Stress und im realistischen Großtiermodell effektiv zu trainieren. Denkbar wäre daher auch ein zweistufiges Kurskonzept mit einfacheren und technisch weniger anspruchsvollen „Anfängerkursen“ für junge Fachärzte [21]. Vor dem Hintergrund dieser und anderer aktueller Studienergebnisse sollte in Analogie zu international anerkannten Kursformaten wie ATLS® eine regelhafte Wiederholung solcher Kursteilnahmen erwogen werden. Demnach empfehlen die Autoren den Kurs nach 4 Jahren mit einer Frist von einem Jahr nach Ablauf zu wiederholen.

### Zukünftige Finanzierung und Bündelung der Kursformate

Die effektiven Kosten eines Kursplatzes sind mit ca. 2000 € vergleichsweise hoch und beruhen auf dem komplexen Kursaufbau und dem hohen Materialverbrauch. So ist die Organisation und Durchführung des Kurses trotz der vergleichsweise hohen angesetzten Kursgebühren zuletzt nicht kostendeckend. Da ein Drittel der Kursteilnehmer ihre Teilnahmegebühren selbst zahlt, wurde auf eine weitere Steigerung der Kursgebühr verzichtet. Entsprechend erhielten zwei Drittel eine finanzielle Unterstützung durch den Arbeitgeber. Die Inhalte und Fähigkeitsvermittlungen des Kurses zielen aber nicht auf den klinischen Alltag und die Karriere bestimmende Elektiveingriffe, sondern auf notfallchirurgisch lebensrettende Fertigkeiten ab. Die chirurgische Fort- und Weiterbildung dieser Fähigkeiten stellt daher, wie so oft im Notfallwesen, auch eine gesellschaftliche Verantwortung dar. Müssen Chirurgen aus eigener Initiative und auf eigene Kosten diese Verantwortung sicherstellen?

Die Umfrage beantwortet das eindeutig. So würden zwei Drittel der Kursteilnehmer erwarten, dass sich Bundesländer und Kliniken die Kursgebühren teilen. Immerhin 20 % sehen allein die Bundesländer in der Verantwortung der Kursfinanzierung.

Seit vielen Jahren ist über die IATSIC (International Association for Trauma Surgery and Intensive Care) das in Tab. 3 aufgeführte, inhaltlich ähnliche, internationale Kursformat (DSTC™; Definitive Surgical Trauma Care) aufgelegt und auch in Deutschland etabliert. Trotz unterschiedlicher Facetten versuchen beide Kursformate, der DGAV- oder auch der DSTC™-Kurs, die Weiterbildungs- und Erfahrungslücken in der Versorgung von Höhlenverletzungen zu kompensieren. Demnach bilden verschiedene Kursformate derzeit in Deutschland notfallchirurgische Fähigkeiten mit variierenden Schwerpunkten aus.

Eine Bündelung der Inhalte ist vor dem Hintergrund des Bedarfs einer fachgebietsübergreifenden kurrikularen notfallchirurgischen Fort- und Weiterbildung sinnvoll und notwendig [18].

## Fazit für die Praxis

- Änderungen der WBO und die geringe Anzahl der operativ zu versorgenden Körperhöhlenverletzungen erfordern eine Ergänzung der Weiterbildung um geeignete Kursformate im Bereich lebensrettende Notfallchirurgie.
- Kursformate, die lebensrettende notfallchirurgische Prinzipien und Fertigkeiten vermitteln sind weltweit etabliert und verschaffen den Teilnehmern nachweislich Sicherheit für den Ernstfall.
- Sie sollten ebenso wie beispielweise das ATLS®- oder ETC® Format nach 4–5 Jahren wiederholt werden.
- Die Fortbildung notfallchirurgischer Fähigkeiten und Kenntnisse liegt im gesellschaftlichen Interesse und zumindest anteilig in ihrer Verantwortung.
- Eine Bündelung der Kursformate unterschiedlicher Fachgesellschaften in Deutschland ist vor dem Hintergrund einer fachgebietsübergreifenden kurrikularen notfallchirurgischen Fortbildung sinnvoll und notwendig.
